# SERUM VALUES OF ALKALINE PHOSPHATASE AND LACTATE DEHYDROGENASE IN EWING'S SARCOMA

**DOI:** 10.1590/1413-785220162404161312

**Published:** 2016

**Authors:** André Mathias Baptista, Juan Pablo Zumárraga, Renan Pires Negrão dos Santos, Guilherme de Oliveira Haubert, Olavo Pires de Camargo

**Affiliations:** 1. Universidade de São Paulo, Faculdade de Medicina, Hospital das Clínicas, Institute of Orthopedics and Traumatology, São Paulo, SP, Brazil.; 2. Hospital Regional do Gama, Orthopedics and Traumatology Unit, Brasília, DF, Brazil.; 3. Universidade de São Paulo, Faculdade de Medicina, Department of Orthopedics and Traumatology, São Paulo, SP, Brazil.

**Keywords:** Sarcoma, Ewing. Alkaline phosphatase. L-Lactate dehydrogenase. Tumor necrosis factor-alpha. Drug therapy. Prognosis.

## Abstract

**Objective:**

To study the relationship between the serum levels of alkaline phosphatase (AP) and lactate dehydrogenase (LDH), and the percentage of tumor necrosis (TN) in patients with Ewing´s Sarcoma (ES)***.***

**Methods:**

This is a case series with retrospective evaluation of patients with diagnosis of ES divided into 2 groups: Group 1, patients whose serum levels of alkaline phosphatase (ALP) and lactate dehydrogenase (LDH) were obtained in the staging phase before preoperative chemotherapy (CT), and Group 2, patients whose values were measured after completion of the preoperative CT. The percentage of tumor necrosis (TN) of surgical specimens extracted in surgery was also evaluated***.***

**Results:**

Eighty four medical records from 1995 to 2015 were included. Both AP as LDH decreased in the patients studied, the pre CT value being higher than the post CT value. The average decrease of LHD was 272.95 U/L and AP was 10.17 U/L. The average tumor necrosis was 65.12 %. There was no statistical correlation between serums levels and the tumor necrosis percentage***.***

**Conclusion:**

The serum levels values of AP and LDH are not predictors for chemotherapy-induced necrosis in patients with ES. ***Level of Evidence IV, Case Series.***

## INTRODUCTION

Ewing's sarcoma (ES) is the second most frequent primary tumor of non-hematopoietic bone, with a peak incidence in the second decade of life. With an estimated incidence of three cases per million, there is a significant association with ethnic groups, being more common in white men. If no treatment is offered, the disease is invariably fatal due to its aggressive local behavior and spread, especially to the lungs.^1^ ^-^ ^4^

The survival rate has improved considerably with the introduction and evolution of chemotherapy. However, the identification of prognostic factors for ES has been a major problem for orthopedic oncology centers. The age, gender, anatomical location, tumor size and serum values of LDH and necrosis are some of the prognostic factors studied in ES.^5^ ^,^ ^6^

ES's response to chemotherapy is considered one of the most important indicators of overall patient survival. This response is measured by the percentage of necrosis found in the resected tumor reported by the pathologist.^7^ ^,^ ^8^

## METHODS

The study was approved by the Ethics Committee of the Department of Orthopedics and Traumatology, of IOT-HC-FMUSP under number 1172/2016. It is a retrospective study based on medical records of patients diagnosed with ES who were treated by the Orthopedic Oncology Group of the Institute of Orthopedics and Traumatology, Hospital das Clinicas, Faculdade de Medicina, Universidade de São Paulo (IOT-HC-FMUSP), from 1995 until the beginning of September 2015.

Patients diagnosed with ES by anatomopathological exam, which had recorded the serum levels of AP and LDH before and after preoperative chemotherapy and the percentage of tumor necrosis in surgical specimens after chemotherapy, were included in the study.

Two hundred and ninety two records of patients with pathological diagnosis of ES were studied. We obtained from the medical records the following epidemiological information: gender, anatomical location of the tumor, histological diagnosis and the patient's age. Serum values of AP and LDH before and after preoperative chemotherapy, as well as the percentage of tumor necrosis of each resected specimen were also recorded. Of the 292 original records, 208 were excluded because they did not present complete data for further analysis. Therefore, 84 patients' records were included in the study. Serum values ​​of AP and LDH of the patients included were obtained before and after completion of preoperative chemotherapy. The tumor necrosis percentage of each resected specimen was also collected.

Regarding the study sample, the mean age of patients was 18.2±11.2 years old. Most patients were males (59.5%) and 40.5% were females. The most frequent tumor location was the femur (34.5%), followed by the humerus, (9.5%). We classified AP and LDH results into two groups: Group 1, patients with normal values of AP and LDH, according to the reference values from HC-FMUSP Laboratory (LDH between 24 and 480U/L, AP between 35 and 130U/L, analysis performed by a COBAS modular machine Roche/Hitachi). In group 2, we had patients with enzyme levels above the normal ranges. The classification used to group the serologic values research was adapted from Bramer et al.^9^ We reported the percentage of tumor necrosis described by pathologists, obtained from the parts extracted at surgeries performed after chemotherapy. This percentage was used according to the index of tumor necrosis described by Huvos.^8^ Data were expressed as mean and standard deviation. 

### Statistical Analysis

In order to verify the correlation between the percentage of tumor necrosis and pre- and post-chemotherapy serum values of the enzymes, or their variation, the following statistical analyzes were made: the nominal characteristics of patients were described with use of relative absolute frequencies. The ages of the patients were described as mean and standard deviation.^10^ AP and LDH values and their variations with chemotherapy, as well as the percentage of tumor necrosis in surgical specimens were described with the use of summary measurements (mean, standard deviation, median, minimum and maximum). Correlations were calculated between the percentage of tumor necrosis of surgical specimens and serum values of the enzymes at pre-chemotherapy, post-chemotherapy and their variation (post -pre) and between both enzymes, using the Spearman correlation to verify the correlation between them. The tests were performed at the 5% significance level. The softwares used for the statistical analysis were SPSS 20.0 (Statistical Package for Social Science for Windows version 20.0) and Microsoft Excel 2008.

## RESULTS

Both AP as LDH levels decreased in our patients, when comparing the pre-chemotherapy value to the post-chemotherapy. The mean difference of AP obtained between pre-chemotherapy and post-chemotherapy was 10.17 U/L, and the mean difference between the values of the LDH pre-chemotherapy and post-chemotherapy was 272.95 U/L. The mean value of the percentage of tumor necrosis was 65.12±27.686%. The values ​​ranged between 0% and 100% tumor necrosis, respectively. (Table 1)

**Table 1:** Description of enzymes and its level variations with chemotherapy and percentage of tumor necrosis on each surgical specimens.

Variables	Mean	St. Dev.	Median	Minimum	Maximum	N	*p*
AP pre	171.85	88.62	144.5	33	456	84	0.348
AP post	161.68	95.17	133.0	42	579	84	
Variation of AP (post - pre)	-10.17	102.33	-8.5	-249	483	84	
LDH pre	884.54	983.29	544.0	116	5948	84	0.001
LDH post	611.58	461.49	478.5	181	2568	84	
Variation of LDH (post - pre)	-272.95	808.74	-74.5	-4075	1375	84	
Huvos	65.12	27.686	68.50	0	100	84

AP: alkaline phosphatase; LDH: lactate dehydrogenase; St. Dev: Standard deviation; N: number.

**Figure 1:**
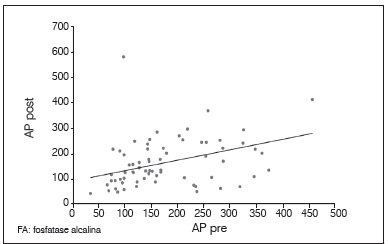
Dispersion diagram between levels of AP pre and post-chemotherapy.

**Figure 2:**
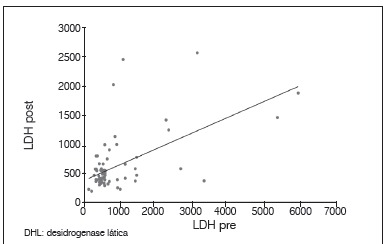
Dispersion diagram between levels of LDH pre and post-chemotherapy.

**Figure 3:**
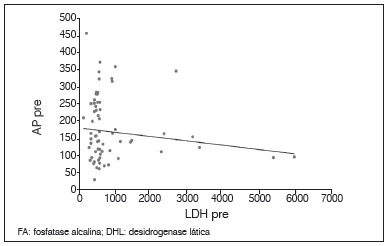
Dispersion diagram between levels of AP and LDH pre-chemotherapy.

**Figure 4:**
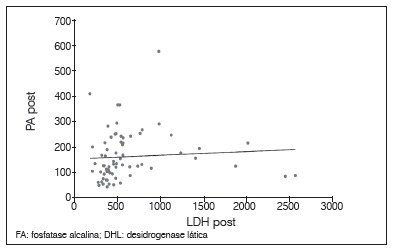
Dispersion diagram between levels of PA and LDH post-chemotherapy.

**Figure 5:**
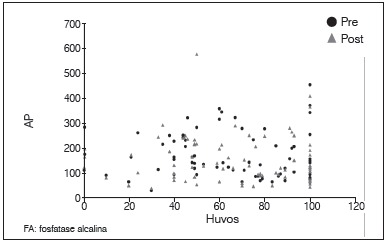
Dispersion diagram between percentage of tumor necrosis and AP levels pre and post-chemotherapy.

**Figure 6:**
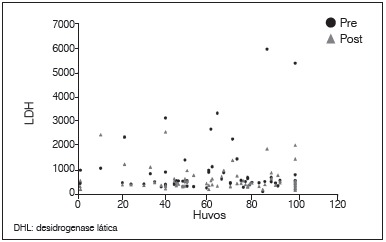
Dispersion diagram between percentage of tumor necrosis and LDH levels pre and post-chemotherapy.

There was no statistically significant correlation between tumor necrosis and the levels of AP and LDH. The lack of relationship was observed with both the pre and post-chemotherapy levels, as with post-chemotherapy values; we did not obtain a correlation with enzyme alteration after chemotherapy (*p*<0.05). Between AP and LDH there was a direct correlation at each time point, as well as the relationship between pre and post-chemotherapy values for the same enzyme (p <0.05). These results are shown in Table 2 and Figures 1-6.

**Table 2:** Results of correlation of the percentage of tumor necrosis with the values of LDH and AP, and between the enzyme levels at every moment of evaluation and its variations after chemotherapy.

Correlation		Huvos	AP pre	AP post	Variation of AP (post - pre)	LDH pre	LDH post
AP pre	r	-0.162					
	p	0.141					
	n	84					
AP post	r	-0.013	0.462				
	p	0.906	<0.001				
		84	84				
Variation of AP (post - pre)	r	0.168	-0.453	0.493			
	p	0.127	<0.001	<0.001			
	n	84	84	84			
LDH pre	r	0.025	-0.094	0.128	0.242		
	p	0.823	0.395	0.247	0.026		
	n	84	84	84	84		
LDH post	r	0.046	-0.111	0.322	0.404	0.341	
	p	0.678	0.315	0.003	<0.001	0.001	
		84	84	84	84	84	
Variation of LDH (post - pre)	r	-0.024	0.003	0.132	0.067	-0.649	0.260
	p	0.831	0.981	0.233	0.548	<0.001	0.017
	n	84	84	84	84	84	84

AP: alkaline phosphatase; LDH: lactate dehydrogenase; N: number.

## DISCUSSION

One of the main prognostic factors for ES is tumor necrosis found in the surgical specimen. However, this information can only be obtained after its resection. If it were possible to identify a prognostic factor before surgery, it could significantly change the surgical option.

The main objective of this study was to determine whether there is a correlation between serum values of AP and LDH and the percentage of tumor necrosis in the tumor extracted after chemotherapy in patients diagnosed with ES.

Ferrari et al.^11^ used the reported pre-chemotherapy levels of LDH as a prognostic factor for ES in adolescents, in a study including 121 patients between 1991 and 2005. They concluded that patients showing a positive response to treatment had normal LDH levels before starting chemotherapy. Bramer et al.^9^ studied 89 adult patients without reported metastasis. They concluded that patients who had a decrease in serum levels of AP after preoperative chemotherapy had a better survival prognosis. In the present study, however, we concluded that there was no correlation between post-chemotherapy serum levels of AP and LDH and the percentage of reported tumor necrosis using the Huvos index, indicating that there is also no correlation of serum levels of the enzymes and the patients' prognosis.

In a study published by Bacci et al.,^12^ 888 patients with ES were evaluated between 1983 and 2006. They used the serum values of LDH as a prognostic factor for metastasis at diagnosis. The authors concluded that patients with elevated serum levels of LDH had a worse prognosis for survival when compared to patients with normal levels. The ES in patients with elevated LDH levels at diagnosis showed a particularly aggressive behavior. 

In another study by Bacci et al.,^13^ they used the serum levels of LDH as a prognostic factor in patients with ES. This study included 579 patients with normal and elevated LDH levels. The conclusion was that the serum level of LDH used as an isolated factor did not correlate to the patients' prognosis.

Gläubiger et al.^14^ published a study in which they analyzed the importance of LDH as prognostic factor for ES progression. One hundred and seventeen patients were enrolled. Unlike ours, this study only used pre-chemotherapy serum levels. They concluded that the use of this serum marker is an important factor in ES prognosis; it has been described that patients with normal LDH levels showed a favorable prognosis as compared to those who had high levels.

Givens et al.^15^ published a study of 87 patients diagnosed with ES, of which 75% had a follow-up for over 20 years. They reported that the life expectancy of these patients was more favorable when the disease appeared before 10 years of age. As part of monitoring, they measured the serum levels of LDH. They reported that serum LDH levels were not considered an important prognostic factor for the percentage of tumor necrosis or survival, as well as the results of the present study.

In two studies published by Seddon et al.^16^ and Craft et al.,^17^ prognostic factors such as age, gender, tumor location, response to chemotherapy and serum levels of LDH were analyzed in patients with ES, including those with metastases. The response to chemotherapy and serum levels of LDH had a prognostic value for survival. However, the authors did not study the relationship between serum values of AP and LDH with the percentage of tumor necrosis, which was the main objective of our study. They also reported that patients with elevated LDH levels had a diminished survival as compared to patients with normal levels.

The literature found on this subject did not compare the measurements of the two enzymes, it only reported LDH levels. We also found no studies that compare pre-chemotherapy and post-chemotherapy levels of the enzymes. What it is reported in the literature are the pre-chemotherapy levels of LDH. Another data that has not been found is the percentage of tumor necrosis on surgical specimens and its relationship with the enzymes levels, which was a main goal of our research.

Finally, we believe that this is the only study that tested the correlation between pre and post-chemotherapy serum values ​​of AP and LDH with the percentage of tumor necrosis; we did not observe any correlation between them.

## CONCLUSION 

Serum levels of AP and LDH have no correlation with the percentage of tumor necrosis in cases of Ewing's sarcoma.
